# Reducing N6AMT1-mediated 6mA DNA modification promotes breast tumor progression via transcriptional repressing cell cycle inhibitors

**DOI:** 10.1038/s41419-022-04661-8

**Published:** 2022-03-07

**Authors:** Jiongyu Chen, Yixuan Zhuang, Ping Wang, Jinfeng Ning, Wei Liu, Yiteng Huang, Xueqiong Lin, Lin Peng, Donghong Zhang

**Affiliations:** 1 Central Laboratory, Cancer Hospital of Shantou University Medical College, 7 Raoping Road, Shantou, Guangdong 515041 China; 2 Guangdong Provincial Key Laboratory of Breast Cancer Diagnosis and Treatment, Cancer Hospital of Shantou University Medical College, Shantou, Guangdong 515041 China; 3 Department of Pathology, Cancer Hospital of Shantou University Medical College, Shantou, Guangdong 515041 China; 4 School of Medical Imaging, Tianjin Medical University, Tianjin, 300203 China; 5 Thoracic Department of Harbin Medical University Cancer Hospital, 150 Haping Road, Harbin, 150040 China; 6 Fourth Department of Medical Oncology, Harbin Medical University Cancer Hospital, 150 Haping Road, Harbin, 150040 China; 7 Health Care Center, First Affiliated Hospital of Shantou University Medical College, 52 Southern Dongxia Road, Shantou, Guangdong 515041 China; 8 Department of Clinical Laboratory, Cancer Hospital of Shantou University Medical College, Shantou, Guangdong 515041 China; 9 Center for Molecular and Translational Medicine, Georgia State University, Research Science Center, 157 Decatur St SE, Atlanta, GA 30303 USA

**Keywords:** Breast cancer, Oncogenesis

## Abstract

DNA N6-methyladenosine (6mA) is a novel epigenetic signaling modification in humans and has been implicated in the progression and tumorigenesis of several cancers. However, the function and mechanism of 6mA in breast cancer (BC), the most common cancer among women, are unclear. Here, we found that decreases in N6AMT1 correlated with the extent of 6mA in clinical BC tissues and predicted a worse survival of BC patients. Functionally, knockdown of N6AMT1 markedly reduced 6mA in DNA and promoted colony formation and migration of BC cells, whereas overexpression of N6AMT1 had the opposite effect. Moreover, silencing of N6AMT1 reduced 6mA modification and enhanced the growth of BC cells in vitro and tumors in vivo. 6mA immunoprecipitation sequencing (6mA-IP-seq), RNA-seq, 6mA-IP-PCR, and bioinformatics analysis indicated that N6AMT1 was a functional methyltransferase for genomic 6mA DNA modifications and related to gene transcriptional activity. Critical negative regulators of the cell cycle, such as RB1, P21, REST, and TP53 were identified as targets of N6AMT1 in BC. These results suggest N6AMT1 enhances DNA 6mA levels to repress tumor progression via transcriptional regulation of cell cycle inhibitors.

## Background

Breast cancer (BC) is the most frequent cancer type among women and remains the leading cause of cancer-related death worldwide [1]. Approximately, 70–80% of BC patients with early-stage or non-metastatic disease are curable, whereas advanced patients do not attain complete remission with the currently available treatment regimens [2]. The incidence, biology, and survival of BC vary worldwide [3], and multiple factors, such as genetic predisposition, lifestyle, and environmental factors, contribute to the variation. Thus, it is imperative to understand the underlying molecular mechanism of BC biology in detail.

The exact mechanism for BC is unclear, although great progress has been made. Notably, aberrant epigenetic landscapes, such as DNA methylated on cytosine residues at the 5 positions (5mC), histone modification, non-coding RNA, and microRNAs have been repeatedly implicated in BC tumor initiation and progression [4–6]. Our recent study and increasing evidence have shown that the dysregulation of RNA-related N6-methyladenosine (m6A) effectors, including methyltransferases, demethylases, and m6A-binding proteins, is pivotal in BC pathogenesis [7, 8]. Several clinical studies have investigated the efficacy of DNA methyltransferase and histone deacetylase inhibitor administration as monotherapy for BC [5, 9]. However, these epi-drugs have shown limited antitumor efficacy at the maximum tolerated doses, suggesting the unsuitability of this approach.

DNA modification by methylation of adenine residues at the N6 position, to form N6-methyladenine (6mA), has been identified as a novel epigenetic mark in prokaryotes and eukaryotes and plays crucial roles in DNA replication, nucleoid segregation, and gene expression [10, 11]. Recently, DNA 6mA modification has been implicated in human cancer progression and tumorigenesis, specifically in glioblastoma, liver cancer, and gastric cancer [5, 12]. Moreover, biochemical and structural evidence demonstrates that N6AMT1 and ALKBH1 function as a methyltransferase and demethylase of 6mA, respectively [12–15]. However, the role of DNA 6mA in human BC biology remains unknown.

To investigate the regulation, functional role, and underlying mechanism of 6mA DNA modification in the development and progression of BC, we first analyzed 6mA and its regulator levels in clinical BC cohorts. Then, the gain- and loss-of-function of N6AMT1 were used to investigate the cancer proliferation and migration in vitro and in vivo. Furthermore, the underlying mechanism of N6AMT1-mediated 6mA formation was analyzed by 6mA immunoprecipitation sequencing (6mA-IP-seq), and 6mA-IP-qPCR and RNA sequencing. Results show that downregulation of N6AMT1 reduces 6mA levels, resulting in enhanced tumorigenesis and a poorer prognosis of BC.

## Materials and methods

### BC clinical specimens and cell lines

In total, 96 cases of primary BC patients were involved in this study. All patients underwent surgical resection at the Cancer Hospital of Shantou University Medical College, China. BC tumor samples and their adjacent non-tumorous breast (BN) samples were collected immediately after surgery and stored at −80 °C. Thirty-two cases of age-matched normal breast tissues were used. For fresh primary BC tissues, two cohorts of training and validation sample sets were as follows: BC tissue samples (*n* = 41) and normal breast tissue samples (BN, *n* = 32), and BC tissue samples (*n* = 22) and their adjacent non-breast cancerous (NBC) samples. For paraffin sections, 96 BC tumor tissues and 14 normal breast tissue samples were used. Clinical characteristics of the patients were analyzed retrospectively. All cancer patients had not received preoperative anticancer treatment. Informed consent was obtained from each patient.

BC cell lines (BT-549, MDA-MB-231, MDA-MB-453, SKBR3, MCF7, and T47D-7), were obtained from the Cell Bank of the Chinese Academy of Sciences (Shanghai, China). Cells were maintained and cultured in accordance with protocols provided by the Cell Bank. All cell lines were authenticated and confirmed negative for mycoplasma contamination by the providers.

### Establishment of N6AMT1 knockout and overexpressing cells

For siRNA knockdown, siRNA targeting human N6AMT1 and scrambled siRNA (Si-CN) were designed and synthesized by Gene Pharma (Shanghai). SiRNAs (10 nM) were transfected into MDA-MB-453 cells using Lipofectamine RNAiMAX Transfection Reagent (Invitrogen) [16].

For shRNA knockdown, pLKO.1 lentiviral shRNA constructs targeting human N6AMT1 or a control shRNA insert were purchased from Dharmacon. pLKO.1 constructs together with packing and helper plasmids, pLP1, pLP2, and VSVG, were co-transfected into HEK293T cells. Viruses were collected at 72 h after transfection and then used to infect MDA-MB-453 cells with polybrene (8 mg/ml, Sigma). At 48 h after infection, puromycin was added to the culture medium for the selection of infected cells.

For overexpression, pCDH lentiviral vectors expressing N6AMT1 together with delta 8.9 and VSVG were transfected into and packaged in HEK293T cells. Transfection of plasmids was performed using Lipofectamine 3000 (Invitrogen) according to the manufacturer’s instructions. Viruses were collected after transfection and then used to infect SKBR3 cells with polybrene. Puromycin was used for the selection of infected cells.

### Genomic DNA extraction

Genomic DNA was extracted from cancer tissues and cultured cells via a TIANamp Genomic DNA Kit (Tiangen Biotech, China). To exclude contaminating RNA, the genomic DNA was treated with 0.1 mg/ml RNase A. Then, the integration of genomic DNA was confirmed on agarose gel electrophoresis. Ultraviolet spectrophotometry was used to determine the quality and concentration (A260/A280 > 1.8) of the DNA.

### Measurement of 6mA in genomic DNA

6mA in genomic DNA was measured by ELISA and dot-blot assay. ELISA was performed as in our previous report [17, 18]. The MethylFlash 6mA DNA Methylation ELISA Kit (Colorimetric) (EpiGentek, NY) was used according to the manufacturer’s instructions. Each sample was run in triplicate. For dot-blot assays, denatured genomic DNA (2 µl, 100 ng) was spotted onto a nylon membrane, UV-crosslinked (20 s, 1200 J/cm^2^), and blocked in 5% milk for 1 h at room temperature (RT). Then, the membranes were incubated with the 6mA antibody for 4 h, and the secondary antibody for another 1 h at RT. After washing, and the dots was developed and visualized using an ECL detection system (Invitrogen) according to the manufacturer’s protocol. The staining of membranes with methylene blue confirmed the equal DNA loading.

### 6mA-immunoprecipitation (IP) sequencing and 6mA-IP quantitative PCR (qPCR)

Genomic DNA was extracted from MDA-MB-453 cells, and then treated with RNase A. Approximately, 6 µg DNA was sonicated to 200–500 bp and purified. An “**A**” nucleotide and adaptors were sequentially added to the 3′ ends of DNA fragments following the Illumina protocol. Then, the ligated DNA fragments were denatured and immune-precipitated with 3 mg 6mA antibodies (Synaptic Systems). The bound DNA was then treated with proteinase K and purified using a QIAquick PCR Purification Kit (QIAGEN).

For 6mA-IP qPCR, the purified immune-precipitated DNA fragments were used to quantify the enrichment of 6mA in individual genes. Quantitative real-time PCR was performed with a 7300 Real-time PCR System (Applied Biosystems). The primers are listed in Table S1.

For 6mA-IP-seq, six libraries (two biological replicates each for immune-precipitated DNA and their input) were prepared using a NovaSeq 6000 S4 Reagent Kit (300 cycles) according to the manufacturer’s instructions, and DNA was sequenced on an Illumina NovaSeq 6000 platform with 150-bp paired-end reads. Raw data were trimmed using Solexa pipeline software v1.8 (Off-Line Base Caller software, v1.8) and checked with FastQC (v0.11.7). Trimmomatic (V0.32) was used to filter out reads with more than 10% N or more than 50% low-quality base sequences. The high-quality reads were aligned to the human reference genome (hg19) by bowtie2 (v2.3.5) [19]. Duplicated reads were removed by SAMtools (v1.9) [20]. Then, 6mA methylated regions were identified by using MACS version 2 with parameters *p* < 1e−5. The consistent peaks in two replicates identified by bedtools [21] were used for the following analysis. MEME-ChIP was used for motif analysis. Differential 6mA levels between si-N6AMT1 and si-control were identified by the MACS2 bdgdiff function with default parameters. Heatmaps were generated by deepTools [22]. The sequencing data have been deposited into the Gene Expression Omnibus (GEO) under accession number GEO GSE166582.

### RNA sequencing and data analysis

Total RNA (approximately 10 µg) was extracted from triplicate MDA-MB-453 cells, transfected with si-N6AMT1 or si-CN, using an RNeasy Plus Mini kit (QIAGEN). Total RNA with an RNA integrity number of ≥8.0 was used for library construction by a TruSeq Stranded mRNA sample preparation kit (Illumina). Libraries were sequenced on an Illumina HiSeq 4000.

We obtained 151 bp paired-end RNA-seq reads, averaging 23 million read pairs for 3 si-N6AMT1 and 3 si-CN samples. Adapters and low-quality bases in reads were trimmed by Trim Galore (v0.6.5; http://www.bioinformatics.babraham.ac.uk/projects/trim_galore/). We employed kallisto (v0.46.0) [23] to determine the read count for each transcript, and quantified transcript abundance as transcripts per kilobase per million reads mapped (TPM), using gene annotation in the GENCODE database (v33, hg19) [24]. Then we summed the read counts and TPM of all alternative splicing transcripts of a gene to obtain gene expression levels. We restricted our analysis to 18,556 expressed genes with an average TPM ≥ 1 in either si-N6AMT1 or si-CN samples. DESeq2 (v1.28.1) [25] was used to identify differentially expressed genes (DEGs) (false-discovery rate <0.05). Enriched function terms of DEGs were generated using PANTHER [26]. The RNA-seq data have been deposited in GEO: GSE166582.

### Databases used and KEGG pathway analysis

The transcriptional levels of N6AMT1 and ALKBH1 in BC and normal tissues were determined by analysis of the ONCOMINE database (Curtis Breasts Statistics: 2136 samples [27]) as described [8]. Prognostic results using N6AMT1 and ALKBH1 involved the GEO dataset GSE21653 (266 BC patients) [28] and GSE25066 (508 BC patients) [29], and were obtained using the R2 platform (http://r2.amc.nl). Correlations between N6AMT1 and its target genes were obtained with cBioPortal (www.cbioportal.org) and confirmed with The Cancer Genome Atlas (TCGA) database (1105 samples).

### In vitro colony formation, transwell, and wound healing assays

MDA-MB-453 cell colony formation and transwell and wound healing assays were performed as described previously [30]. Briefly, for colony formation, 400 MDA-MB-453 cells/well were seeded in a 6-well plate on Day 0. After one week, colonies were fixed with methanol and stained with 0.1% crystal violet for 10 min. Then, colonies were photographed using an optical microscope (Olympus, Japan), and colonies larger than 1 mm (>50 cells/clone) were counted. For evaluation of migration, 5 × 10^5^ MDA-MB-453 cells were seeded into the upper chamber of a transwell in a serum-free medium. FBS-containing media was used as the chemo-attractant and added to the bottom chamber. After 24 h, cells that migrated to the lower side of the membrane were fixed with 4% paraformaldehyde and stained with crystal violet after cells on the upper chamber were gently wiped off. Images of stained cells were captured and counted in five random fields. Each experiment was independently repeated three times.

### Immunohistochemical (IHC) staining

Breast tissue slides were routinely deparaffinized and rehydrated. Then, antigen retrieval was performed, followed by 3% H_2_O_2_ treatment. Then, slides were stained with anti-N6AMT1 antibody (1:200, Synaptic Systems) or anti-ALKBH1 (1:200, Synaptic Systems) overnight at 4 °C. After washing, sections were incubated with secondary HRP (horseradish peroxidase)-conjugated anti-rabbit antibody, followed by staining with a diaminobenzidine detection kit (Gene Tech, China) and hematoxylin. Each tissue section was qualitatively analyzed using a relative scale from − (negative) to + (medium) and ++ (strong), then semi-quantified using a histoscore (H-score) as previously reported [31]. The results were evaluated by two independent pathologists.

### Western blotting

Total cellular protein was lysed with a radioimmunoprecipitation buffer containing a proteinase inhibitor cocktail. Cell extracts (20 µg each) were resolved on 10% sodium dodecyl sulfate-polyacrylamide gel electrophoresis and transferred onto polyvinylidene fluoride membranes. After blocking with 5% non-fat milk and incubating with primary antibodies (N6AMT1 (1:500, Synaptic Systems), ALKBH1 (1:500, Synaptic Systems) or β-ACTIN (1:2000, Abcam)), then incubated with secondary antibodies and developed using an ECL detection system (Invitrogen) according to the manufacturer’s protocol.

### RNA isolation and RT-PCR

Total RNA was extracted from cells or tissues using an RNAsimple Total RNA Kit (TIAN GEN) and reverse-transcribed using SuperScript III Reverse Transcriptase (Invitrogen). qRT-PCR was performed using TB Green Premix EX Taq II (TAKARA) and a 7300 Real-time PCR System (Applied Biosystems). PCR products were assessed by melting curve analysis. Relative mRNA levels of the target gene were calculated by the 2^−ΔΔCt^ method. Beta-actin was used as an internal control for the normalization. All primers used in this study are listed in Table S1.

### In vivo subcutaneous tumor cell implantation model

Six-week-old female severe combined immunodeficiency mice were provided by the Shanghai Laboratory Animal Center (SLAC) (China). After being raised in a pathogen-free animal laboratory for two weeks, mice were randomly divided into a control or experimental group (*n* = 9/each group). Then, 4 × 10^6^ stably infected shRNA-N6AMT1 or shRNA-control MDA-MB-453 cells were suspended in 100 µl serum-free medium and Matrigel (BD Biosciences, USA), at a 1:1 ratio, and injected subcutaneously. After 2 weeks, the tumor volumes were measured by with a Vernier caliper every three days and calculated using the following formula: volume = (length × width^2^)/2. When tumor volumes reached 1000 mm^3^, mice were euthanized and tumors were weighed.

### Statistical analysis

All experiments were repeated at least three times. Statistical analyses were carried out using SPSS 20.0 software. All data are presented as the mean ± standard deviation except where stated otherwise. Unpaired or paired two-tailed Student’s *t*-tests were applied to compare data between two groups, and one-way ANOVA was used for multiple comparisons. A chi-square test was used to evaluate the statistical significance of differences in IHC scores between BC and adjacent noncancerous samples. Correlation between two groups was determined by Spearman’s test. Survival curves were obtained using the Kaplan–Meier method and compared by the log-rank test. A *p < 0.05, **p < 0.01, or ***p < 0.001 was considered statistically significant.

## Results

### Reduction of N6AMT1 correlates with reduced DNA 6mA, enhanced tumor progression, and poor prognosis of BC patients

**Fig. 1:**
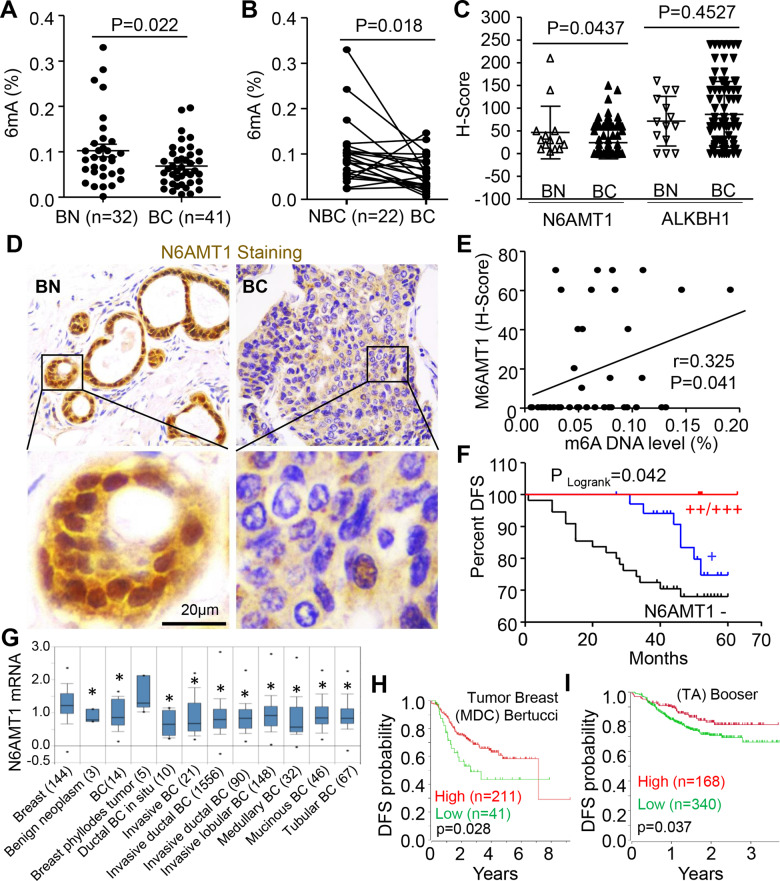
Reduction of N6AMT1 and DNA 6mA levels correlates with poor prognosis of BC patients. **A**, **B** Levels of the global N6-methyladenine (6mA) in DNA were assessed via ELISA in primary breast cancer tissues (BC), normal breast tissues (BN), and adjacent non-breast cancerous tissues (NBC). Unpaired and paired *t*-tests were performed in (**A**) and (**B**), respectively. **C** Statistical analysis (Student’s *t*-test) of N6AMT1 and ALKBH1 protein between BC (*n* = 96) and NC (*n* = 14). **D** IHC staining for N6AMT1 in BC and BN samples. **E** Correlation between N6AMT1 protein and DNA 6mA levels in BC tissue was analyzed by Spearman’s *t*-test. **F** Kaplan–Meier survival curves of the disease-free survival (DFS) based on N6AMT1 expression level. **G** Box plots showing N6AMT1 mRNA expression from the ONCOMINE database analysis of breast statistics. *P < 0.05 vs. normal breast tissues. **H**, **I** Prognostic value of N6AMT1 mRNA levels in BC patients from the studies of Tumor Breast (MDC) Bertucci and (Taxane–Anthracycline) Booser. Statistical analysis was done using the log-rank test.

To assess the clinical significance of DNA 6mA in BC development, we performed an ELISA assay for 6mA on our two BC cohorts. For the training sample set, the level of 6mA in BC tissue was reduced compared to the random normal breast tissue (BN) (Fig. 1A). For the validation sample set, a similar pattern of 6mA was found in BC compared to their adjacent non-breast cancerous normal tissue (NBC) (Fig. 1B). IHC staining and quantification indicated that N6AMT1 was found to be moderately expressed in the nucleus in normal mucosa tissues but was rarely detected in the nucleus of BC tissues (Fig. 1C, D). The decrease in N6AMT1 expression positively correlated with 6mA levels in BC tissues (Fig. 1E) and tumor differentiation (Table 1), and inversely correlated with tumor, node and metastasis stage, tumor size, and death. On the other hand, the expression of ALKBH1, the demethylase for 6mA, was unchanged between BC and BN (Fig. S1A). Kaplan–Meier survival analysis further suggested that BC patients with lower N6AMT1 protein levels had worse disease-free survival than those with high expression (Fig. 1F). To confirm the clinical findings, we queried a breast statistics database of 2136 samples [27] and found decreased N6AMT1, but not ALKBH1 mRNA expression in nearly all types of breast tumors compared with normal breast (Figs. 1G, S1B). The decrease in N6AMT1 mRNA level was significantly correlated with the worse outcome from two cohorts in the Kaplan–Meier plotter (Fig. 1H, I) and bc-GenExMiner (bc-GenExMiner v4.0) (Fig. S2) databases. ALKBH1 expression was not prognostic (Fig. S1C). These results suggest a reduction of N6AMT1 correlates with DNA 6mA, tumor progression, and poor prognosis of BC patients.

**Table 1:** Correlation of N6AMT1 protein expression and clinical characteristics of patients with breast cancer.

Characteristics		N6AMT1−	N6AMT1+	*R* value	*P* value
6mA%	<0.3	25	10	7.590	**0.006**
	>0.3	14	22		
ALKBH1	−/+	26	13	3.337	0.068
	++/+++	18	21		
Age, years	<50	25	16	0.02	0.887
	≥50	16	11		
Menopause	No	22	21	1.073	0.300
	Yes	22	13		
Tumor size, cm	<13	17	21	4.11	**0.043**
	≥13	27	13		
TNM	I	6	12	6.38	**0.041**
	II	22	13		
	III + IV	16	9		
Stage	I	6	10	3.190	0.203
	II	18	13		
	III + IV	20	11		
Differentiation	I	5	10	6.29	**0.043**
	II	14	10		
	III	25	14		
Lymph node metastasis	No	19	12	0.498	0.480
	Yes	25	22		
Metastasis	No	33	29	1.247	0.264
	Yes	11	5		
KI67 level	<30%	16	16	0.758	0.384
	≥30%	27	18		
Relapse	No	36	29	0.036	0.850
	Yes	7	5		
Death	No	32	31	4.20	**0.040**
	Yes	12	3		

Statistical analysis involved a chi-square test. Bold, *p* < 0.05.

### N6AMT1-mediated DNA 6mA formation represses proliferation and migration of BC cells in vitro

**Fig. 2:**
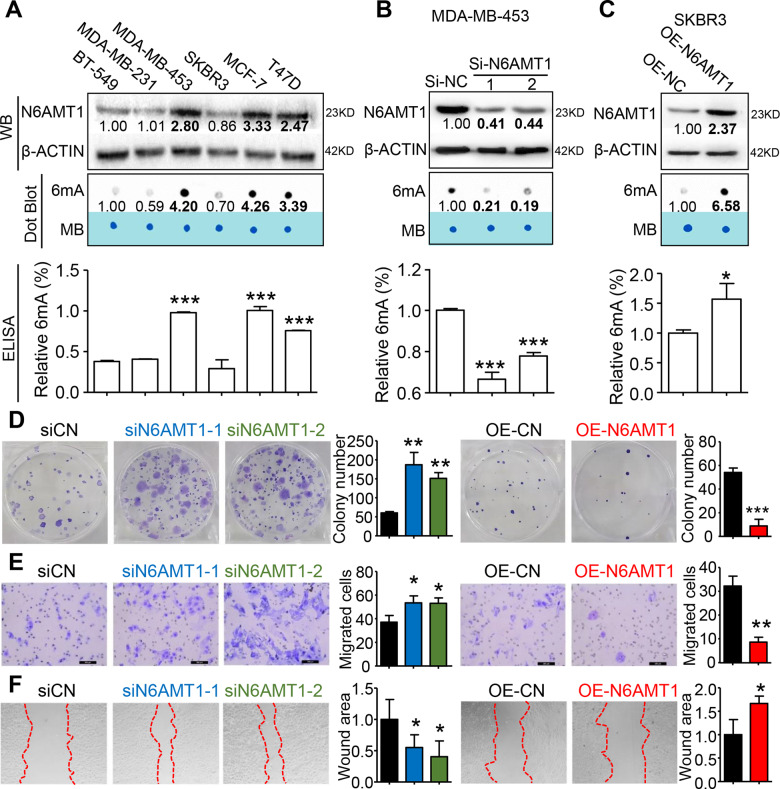
N6AMT1-mediated 6mA modification represses proliferation and migration of BC cells in vitro. **A** Levels of N6AMT1 protein (top panel) and DNA 6mA levels (middle and lower panels) in various breast cancer cells were analyzed by western blot (WB, n = 3), dot-blot (*n* = 4), and ELISA (*n* = 4), respectively. β-ACTIN and methylene blue staining (MB) were used as the loading control for WB and dot-blot assays, respectively. The value is the relative ratio to BT-549 cells. Bold fonts indicate p-values less than 0.05, calculated by one-way ANOVA followed by the Bonferroni test. **B** Levels of N6AMT1 protein and 6mA in MDA-MB-453 cells following siN6AMT1 or si-NC transfection. The value is the relative ratio to si-NC. Bold fonts indicate *p*-values less than 0.05, calculated by one-way ANOVA followed by the Bonferroni test. **C** Levels of N6AMT1 protein and 6mA in SKBR3 cells following lentivirus-N6AMT1 (OE-N6AMT1) or -control (OE-CN) transduction. The value is the relative ratio of OE-NC. Bold fonts indicate p-values less than 0.05, calculated by Student’s *t*-test. **D**–**F**, Knockdown of N6AMT1 enhanced colony formation and migration of MDA-MB-453 cells, whereas N6AMT1 overexpression impaired colony formation and migration of SKBR3 cells. *p < 0.05, **p < 0.01, **p < 0.001 vs. si-CN or OE-CN.

To verify whether N6AMT1 directly regulates DNA 6mA levels in BC, we screened N6AMT1 protein expression in BC cells. N6AMT1 was highly expressed in MDA-MB-453, MCF7, and T47D cells, but low in BT-549, MDA-MB-231, and SKBR3 cells (Fig. 2A). Concomitantly, the level of DNA 6mA methylation displayed a similar pattern by dot-blot and ELISA assays of the BC cells (Fig. 2A). Then, we established N6ATM1 loss and overexpression by transient transfection of MDA-MB-453 and SKBR3 cells, respectively. Western blot analysis indicated successful knockdown and overexpression for the corresponding transfection. N6ATM1 knockdown decreased both N6ATM1 expression and 6mA levels in MDA-MB-453 cells, whereas the converse effect was found with N6ATM1 overexpression in SKBR3 cells (Fig. 2B, C).

Colony formation assays indicated that N6ATM1 knockdown increased the number and size of colonies formed by MDA-MB-453 cells, whereas N6ATM1 overexpression repressed colony formation of SKBR3 cells (Fig. 2D). Similarly, in transwell assays, cell migration was accelerated by silencing and inhibited by overexpressing of N6ATM1 in MDA-MB-453 and SKBR3 cells, respectively (Fig. 2E), as was wound healing (Fig. 2F). These findings support the conclusion that N6AMT1 directly mediates the formation of DNA 6mA and represses colony formation and cell migration of BC cells in vitro.

### Silencing N6AMT1 enhances the growth of BC cells in vivo

**Fig. 3:**
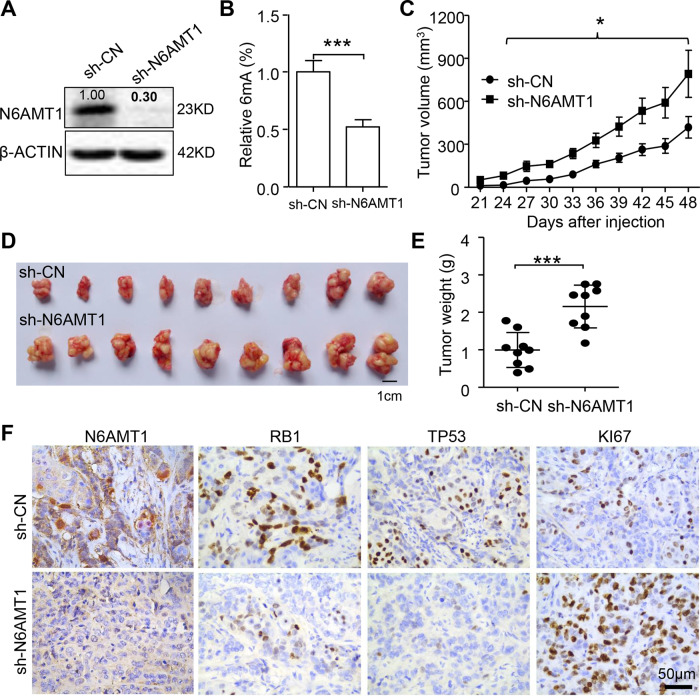
Silencing N6AMT1 reduces DNA 6mA and promotes the growth of BC cells in vivo. **A** Western blot showing N6AMT1 was successfully knocked down by shN6AMT1 lentivirus in MDA-MB-453 cells. The value is the relative ratio to sh-NC. Bold fonts indicate *p*-values less than 0.05, calculated by Student’s *t*-test. **B** Reduction of 6mA in MDA-MB-453 cells following N6AMT1 knockdown, as determined by ELISA. **C**–**E** Tumor growth curves (**C**), representative xenografts (**D**), and tumor weights (**E**) show that N6AMT1 knockdown enhances MDA-MB-453 cell xenograft growth. **F** Representative immunostaining for N6AMT1, RB1, TP53, and Ki67 in xenografts of sh-N6AMT1 and sh-Control tumors.

To evaluate the effect of N6AMT1 in BC in vivo, we established an MDA-MB-453 cell line with a stable knockdown of N6AMT1. Silencing of N6AMT1 reduced DNA 6mA levels in BC cells (Fig. 3A, B). Then, xenograft animal models were generated by injection of nude mice with 10^5^ MDA-MB-453 expressing sh-N6AMT1 or sh-Control. Consistent with the in vitro results, xenografts with silenced N6AMT1 expression grew faster than did the control xenografts when measured at 24 days after injection (Fig. 3C), with sh-N6AMT1 tumor weight being 2-fold greater compared with sh-CN tumors (Fig. 3D, E). In addition, the levels of cell cycle inhibitors (RB1 and TP53) were decreased, while the cell cycling indicator Ki67 was increased in sh-N6AMT1 xenografts compared with sh-Control xenografts (Fig. 3F). Altogether, these data suggest that silencing N6AMT1-mediated DNA 6mA formation enhances the growth of BC cells.

### N6AMT1 is a functional methyltransferase for genomic 6mA in BC cells

**Fig. 4:**
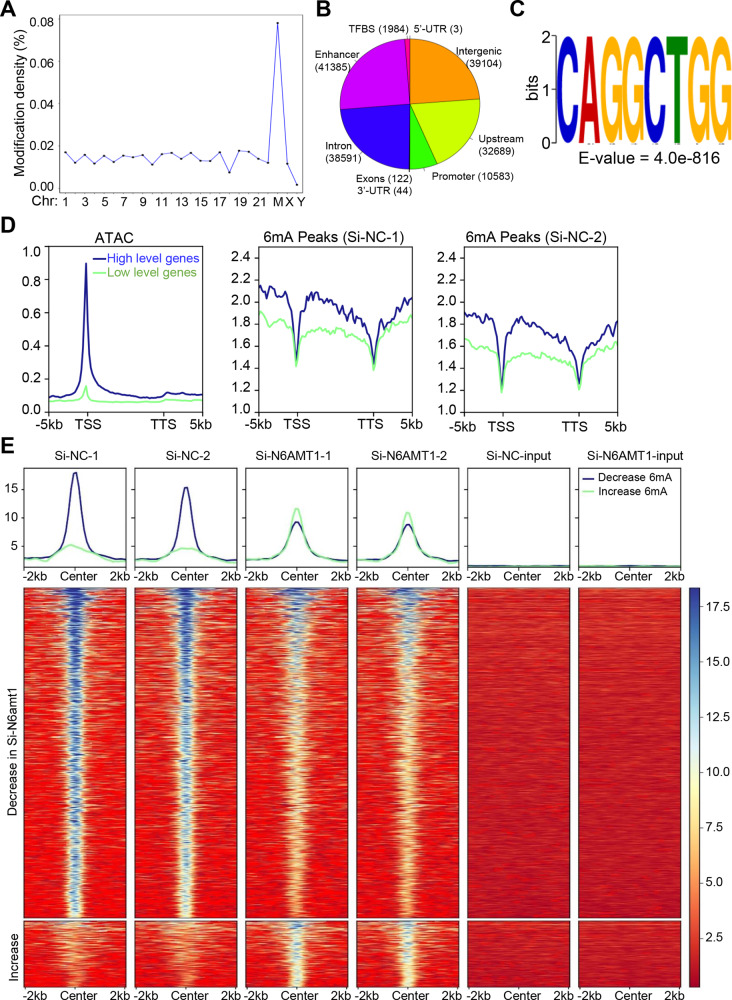
N6AMT1 is a functional methyltransferase for DNA 6mA modification in BC cells. **A**, **B** 6mA distribution across all chromosomes and the functional elements of human genomic DNA. **C** The top motif sequence in 6mA-containing DNA fragment peaks identified by 6mA-IP-seq. **D** Profile plot showing the 6mA-IP-seq and ATAC-seq signals over the 1000 highest/lowest expressed protein-coding genes in MDA-MB-453 cells. **E** Heatmap demonstrating the change of 6mA signal by N6AMT1 knockdown over 6mA peaks. TFBS transcription factor binding sites, TSS transcription start site, TTS transcription termination site.

We further applied 6mA-IP-seq to screen the 6mA modification in the genomic DNA of MDA-MB-453 cells. In total, we identified 17,294 high-confidence 6mA peaks in MDA-MB-453 cells confirming the presence of 6mA modifications. Consistent with a previous study [32], the mitochondrial genome showed the highest 6mA density (>80%) compared to autosomal chromosomes (0–0.7%) (Fig. 4A). The distributions of 6mA modifications were related to regions of different gene functions in the genome [11, 12]. By mapping the genomic distributions of 6mA modifications, we found that about half of the 6mA-containing sites were located in introns and intergenic regions, which account for >85% of the human genome. Another half of the 6 mA sites were located in enhancers and upstream promoter regions, suggesting that 6mA modification may be involved in the regulation of gene expression (Fig. 4B). MEME-ChIP analysis revealed that **CAGG**CTGG was the top-ranking 6mA motif in MDA-MB-453 cells (Fig. 4C), similar to the motif previously discovered in human blood and *Caenorhabditis elegans* genomes [12, 33].

To understand the relationship between DNA 6mA and gene transcription, we quantified gene expression levels by RNA-seq in MDA-MB-453 cells and cataloged the top 1000 highest and 1000 lowest expressed protein-coding genes. ATAC-seq (assay for transposase-accessible chromatin using sequencing) assay data (GEO: GSE157082) supported those two groups of genes, which contained more open and closed promoters, respectively. Notably, plotting of 6mA density showed that the regions with a higher density of 6mA were often located in or near the more highly expressed genes, such as intragenic regions, and upstream and downstream (DNA), but depleted in the transcription start site and the transcription termination site regions themselves (Fig. 4D), suggesting that 6mA modification marks actively transcribed genes in BC cells. To understand the role of N6AMT1 in regulating 6mA modification, we functionally silenced N6AMT1 by si-RNA and conducted 6mA-IP-seq. As expected, the N6AMT1 knockdown led to clear changes in 6mA levels. Of them, about 83% of genomic loci had decreased 6mA levels (1161 peaks), and 17% had increased accessibility (233 peaks) (Fig. 4E). The decreased 6mA peaks were enriched in genes relevant to cancer biologies, such as cell differentiation, cell adhesion, DNA damage stimulus by C-MYC, ErbB, TP53, and estrogen pathways. The increased 6 mA peaks were related to cellular component, organization, protein location, and catabolic process by membrane trafficking, EPHA or insulin pathways (Fig. S3A–D). Taken together, our results reveal that N6AMT1 is a functional methyltransferase for genomic 6mA DNA modification in BC cells.

### Cell cycle inhibitor genes are targets of N6AMT1

**Fig. 5:**
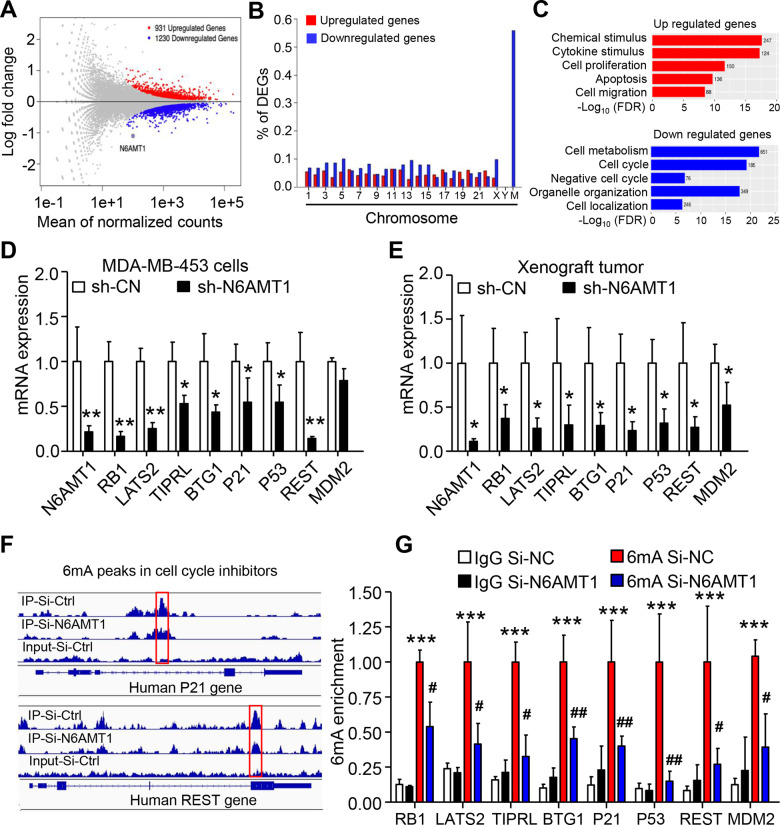
Negative regulators of the cell cycle are targets of N6AMT1 in BC cells. **A** MA (M, log-ratio; A, mean average) plot of differentially expressed genes (DEGs) following N6AMT1 knockdown in MDA-MB-453 cells. **B** Distribution of DEGs across all chromosomes. **C** Bar plots displaying functional enrichment of DEGs following N6AMT1 knockdown. **D**, **E** qRT-PCR verification of decreased cell cycle inhibitors in N6AMT1-silenced MDA-MB-453 cells and the xenograft. *p < 0.05 by the unpaired two-tailed Student’s *t*-test. **F** IGV shows the decrease of piled 6 mA reads of cell cycle inhibitor genes from 6mA-IP-seq in MDA-MB-453 cells by N6AMT1 knockdown. The thinnest blue lines are the introns. The medium and thickest thickness segments are untranslated and translated exons, respectively. **G** 6mA-IP-qPCR assay indicates that silencing N6AMT1 reduced 6mA enrichment. ***P < 0.0001 vs. IgG si-CN. ^#^*P* < 0.05 vs. 6mA si-CN by one-way ANOVA followed by the Bonferroni test. Data are expressed as mean ± standard deviation.

**Fig. 6:**
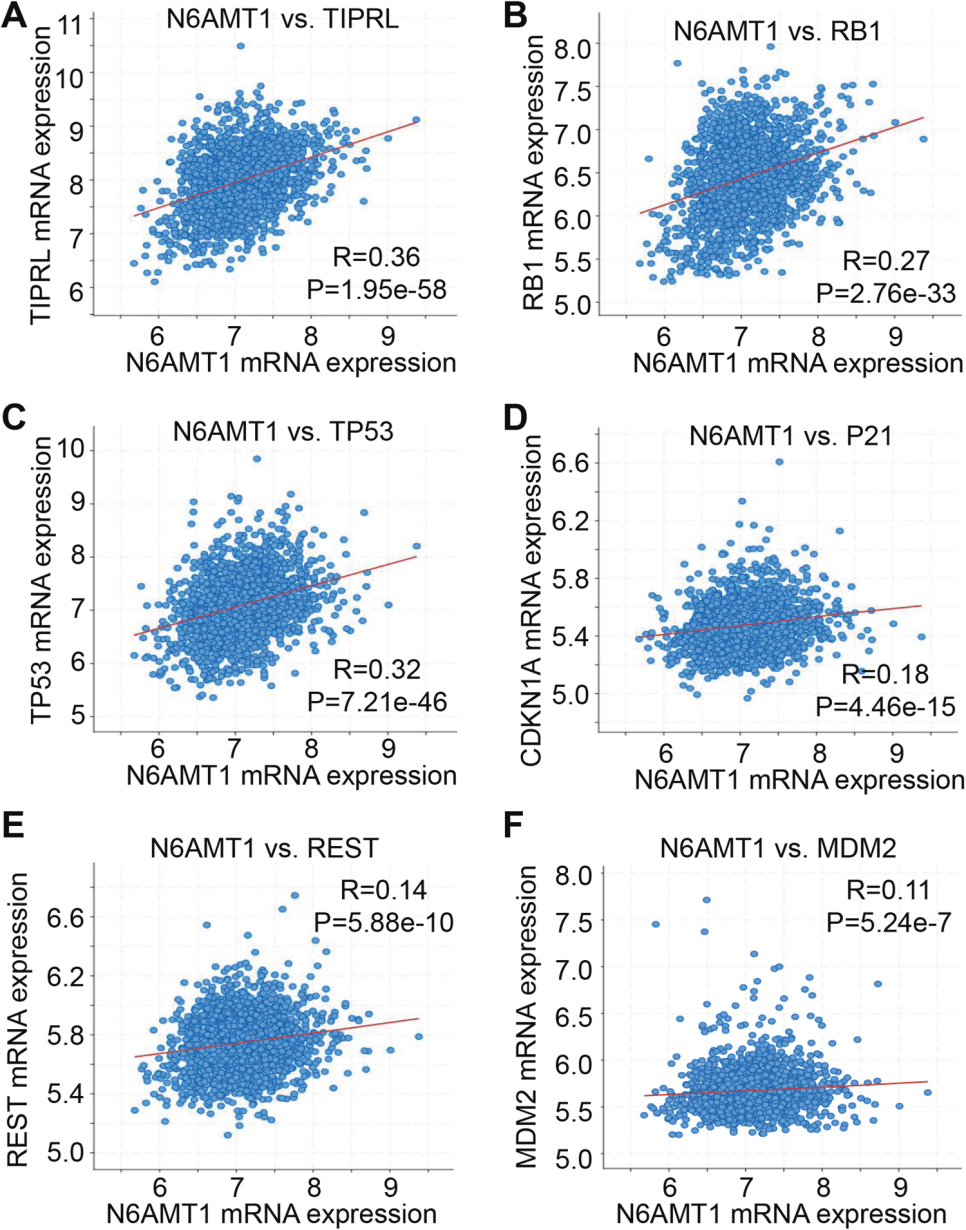
Correlations between N6AMT1 mRNA level and 6 critical negative cell cycle genes expression in 2509 BC tissues in TCGA cohort. Spearman correlation test shows N6AMT1 mRNA expression positively correlates with TIPRL (**A**), RB1 (**B**), TP53 (**C**), P21 (**D**), REST (**E**), and MDM2 (**F**).

To identify the targets of DNA 6mA modification, we conducted RNA-seq in N6ATM1 knockdown (si-N6AMT1) and its control (si-CN) cells. The MA plot indicated that the mRNA levels of 1230 genes were significantly downregulated (including N6AMT1) and 931 genes were upregulated after knocking down N6AMT1 in MDA-MB-453 cells (Fig. 5A). Consistent with 6 mA peaks, downregulated genes were distributed along nearly all chromosomes. The most downregulated genes were in the mitochondrial genome (~55%) (Fig. 5B). To identify the gene pathways regulated by N6AMT1, we performed Gene Ontology (GO) analysis and found the upregulated genes that participated in key oncogenic pathways previously implicated in breast pathogenesis, including chemical and cytokine stimulation, cell proliferation, apoptosis, and migration pathways (Fig. 5C). The downregulated genes were enriched in negative cell cycle control, cell metabolism, organelle organization, and cell location (Fig. 5C). Overall, 456 of the differentially-expressed genes (21%) were closely related to changes in 6mA levels (Fig. S4). Of note, several critical negative regulators of the cell cycle, such as RB1, LAST2, BTG1, TP53, P21, REST, MDM2, and TIPRL, were decreased following N6AMT1 deletion. This finding was further confirmed in cultured and xenograft MDA-MB-453 cells by qRT-PCR (Fig. 5D, E). Interestingly, an Integrative Genomics Viewer (IGV) displayed highly enriched and specific 6mA peaks at cell cycle inhibitor genes that were substantially decreased following N6AMT1 knockdown (Fig. 5F). 6mA-IP-qPCR further confirmed that genes for cell cycle inhibitors exhibited 6 mA changes in their genes, and the modifications were further reduced upon N6AMT1 deficiency (Fig. 5G). The positive correlations between N6AMT1 and TIPRL, RB1, TP53, or CDKN1A mRNA expression were confirmed by the TCGA databases (2509 BC samples) [27, 34], which we obtained from chipportal.org (Fig. 6A–F). Genes for negative regulators of the cell cycle were identified as the targets of N6AMT1 and responded to tumor progression by N6AMT1 knockdown in BC.

## Discussion

We show a novel underlying molecular mechanism for BC biology involving DNA methylation of adenine residues at the N6 position. Briefly, downregulation of N6AMT1, a 6mA methyltransferase, decreases 6mA DNA modification, resulting in progression and worse prognosis of clinical BC patients. Knockdown of N6AMT1 by sh-RNA reduces DNA 6mA levels and enhances the proliferation and migration of BC cells, whereas overexpression of N6AMT1 results in the converse effect. Therefore, N6AMT1 acts as a methyltransferase for DNA 6mA modifications and represses gene expression of critical cell cycle inhibitors, such as RB1 and TP53. This represents the first study for the regulation and function of 6mA DNA modification in BC progression and prognosis.

Methylation at the N6 position of adenine is the most abundant DNA modification and plays essential role in DNA replication, repair, transposition, and transcription in prokaryotes [35, 36]. Increasing evidence indicates that DNA undergoes 6mA modification in both lower and higher eukaryotes [37]. Interestingly, low levels of 6mA (0.1–0.01%) have also been found in mammals by various technologies, such as mass spectrometry, 6mA-IP-sequencing, single-molecule real-time sequencing, dot blot, and immunohistochemistry. Initial studies have demonstrated that 6mA is involved in various biological and disease processes in mammalian genomes, including embryonic stem cell differentiation, osteogenic differentiation, neurologic responses to environmental stress, and tumorigenesis [11, 38–40]. However, the biological function of 6mA in human disease remains largely unclear. Our previous study determined that a reduction of 6mA by elevated ALKBH1 leads to the development of hypertension and atherosclerosis in vivo and in vitro [17, 18]. Mechanistically, 6mA negatively regulates hypoxia-response genes and is involved in vascular remodeling. To date, two recent studies have focused on the involvement of 6mA in cancer. Similar to cardiovascular disease and osteogenic differentiation [17, 18], genomic 6mA levels are reduced in primary gastric and liver cancer tissues, and this 6mA downregulation correlates with increased tumorigenesis [12, 40]. Elevated 6mA has also been found in primary glioblastoma and associated with disease progression [41]. Thus, it is critical to determine the specific roles of 6mA and its regulators for individual cancers.

Limited literature, but consensus evidence indicates that N6AMT1 and ALKBH1 functionally act as a methyltransferase (writer) and demethylase (eraser), respectively, for genomic 6mA modification in specific tumors. Collectively, N6AMT1-mediated 6mA modification inhibits stomach and liver tumorigenesis and metastasis, while ALKBH1 maintains glioblastoma tumor cell viability and stemness properties via 6mA demethylation. Herein, we find N6AMT1 regulation of 6mA contributes to BC development, progression, and prognosis in vivo and in vitro. Secondly, [G/C]AGG was identified as the most prevalent motif for 6 mA modification in BC, consistent with the results found in human blood [12] and the *C. elegans* genome [33], but different from those in the mouse [11] and Chlamydomonas [42]. Thirdly, our current study indicates that 6mA modification marks actively transcribed genes in BC cells, especially for cell cycle inhibitors, including P53 and RB1. Similar regulatory mechanisms have been confirmed in *C. elegans*, *Chlamydomonas reinhardtii*, *fungi*, and *Drosophila melanogaster*, whereas the reverse pattern has been found in mice [11, 33, 43]. These observations suggest that 6mA modification is species-specific and contributes to diverse biological functions and mechanisms. Last, N6AMT1-mediated 6mA modification is notably enriched in breast mitochondria DNA and regulates mitochondrial transcription. This might be related to the cytoplasmic location of N6AMT1 and needs further confirmation. A recent study showed that METTL4 can mediate mammalian mtDNA 6 mA methylation and affect mitochondrial transcription, replication, and activity [32]. It would be worthwhile to exploit more potential mammalian DNA methyltransferases and demethylases to dissect the involvement of DNA 6mA in mammalian development.

In summary, we found that N6AMT1-mediated genomic DNA 6mA modification is reduced in BC and correlates with tumor development, progression, and prognosis in BC patients. Loss- and overexpression of N6AMT1 is involved in the regulation of BC cell proliferation and migration in vitro and in vivo. The most prevalent 6mA motif [G/C]AGG[C/T] marks cell cycle inhibitors and transcriptionally activates gene expression. Our finding of 6mA modifications in BC cells sheds light on epigenetic regulation and function during BC biology.

## Data and materials availability

The 6mA-IP-seq and RNA-seq data have been deposited in the Gene Expression Omnibus (GEO) database (GSE166582). In addition, we also used other previously published ONCOMINE databases of breast statistics. For the determination of the prognostic value of N6AMT1 mRNA levels in BC patients, we used the GEO datasets GSE21653 and GSE25066 from the R2 platform (http://r2.amc.nl). Correlations between N6AMT1 and its target genes were conducted with cBioPortal (www.cbioportal.org) and confirmed with The Cancer Genome Atlas (TCGA) database.

## Supplementary information

SUPPLEMENTAL Figure S1-4

Supplemental Material-Uncropped figures for WB

SUPPLEMENTAL Table S1

Reproducibility checklist
