# Supplementary material for: Reducing N6AMT1-mediated 6mA DNA modification promotes breast tumor progression via transcriptional repressing cell cycle inhibitors
Source: Cell Death Dis. 2022 Mar 7;13(3):216. doi: 10.1038/s41419-022-04661-8 (PMC8901905; doi:10.1038/s41419-022-04661-8)
Supplement: Supplementary file 1 — SUPPLEMENTAL Figure S1-4 [file 41419_2022_4661_MOESM1_ESM.pdf]

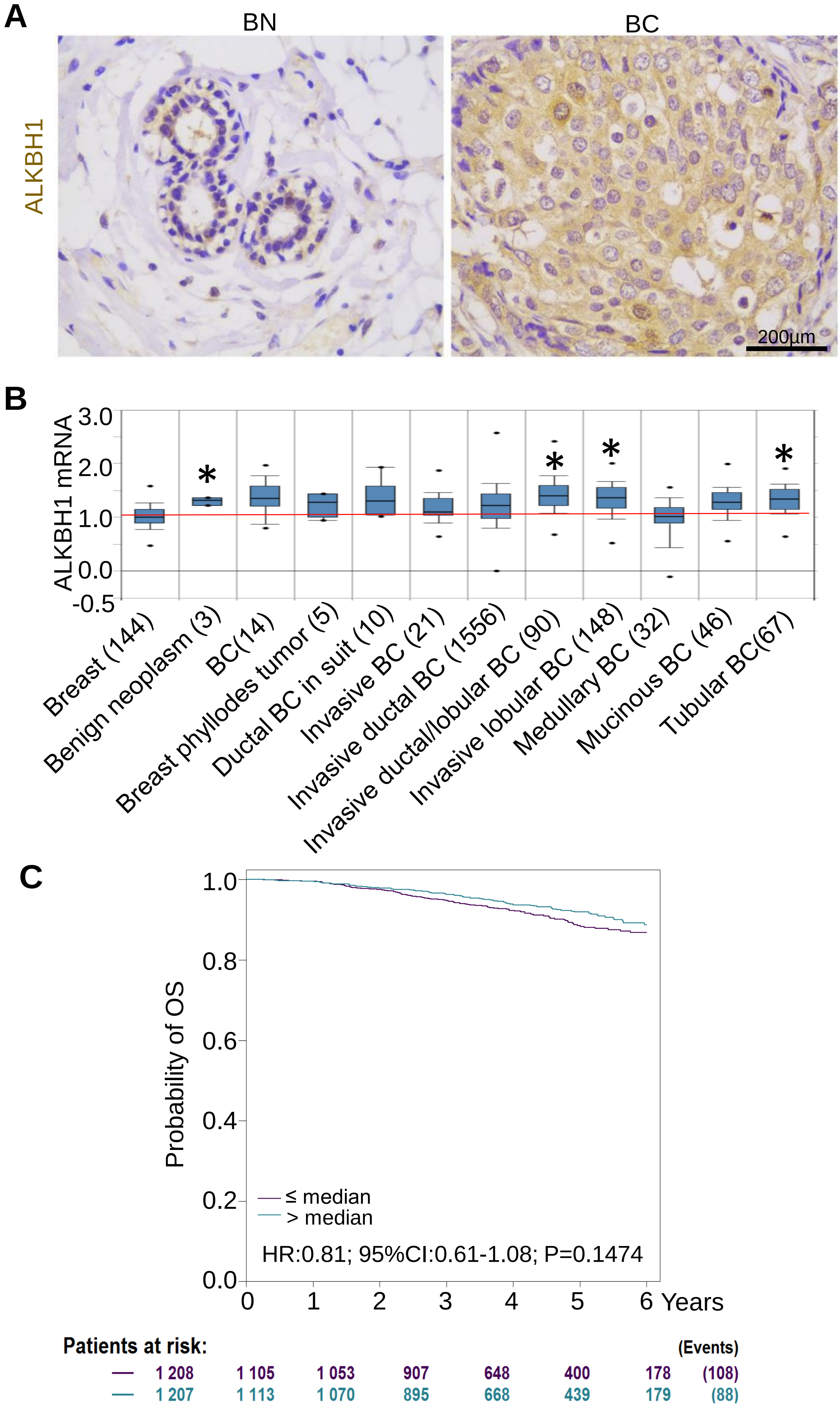

Figure S1. ALKBH1 was dispensable for the prognosis of BC patients. a, Representative immunohistochemistry IHC staining of ALKBH1 in breast cancer tissues (BC) and normal breast tissues (BN) samples. b, Box plots shown ALKBH1 mRNA expression in various kinds of BC from the ONCOMINE database analysis of Curtis breasts statistics. c, The prognostic value of ALKBH1 mRNA level in BC patients from the online studies of the BC (bc-GenExMiner v4.0). *P < 0.05 via normal breast tissues.

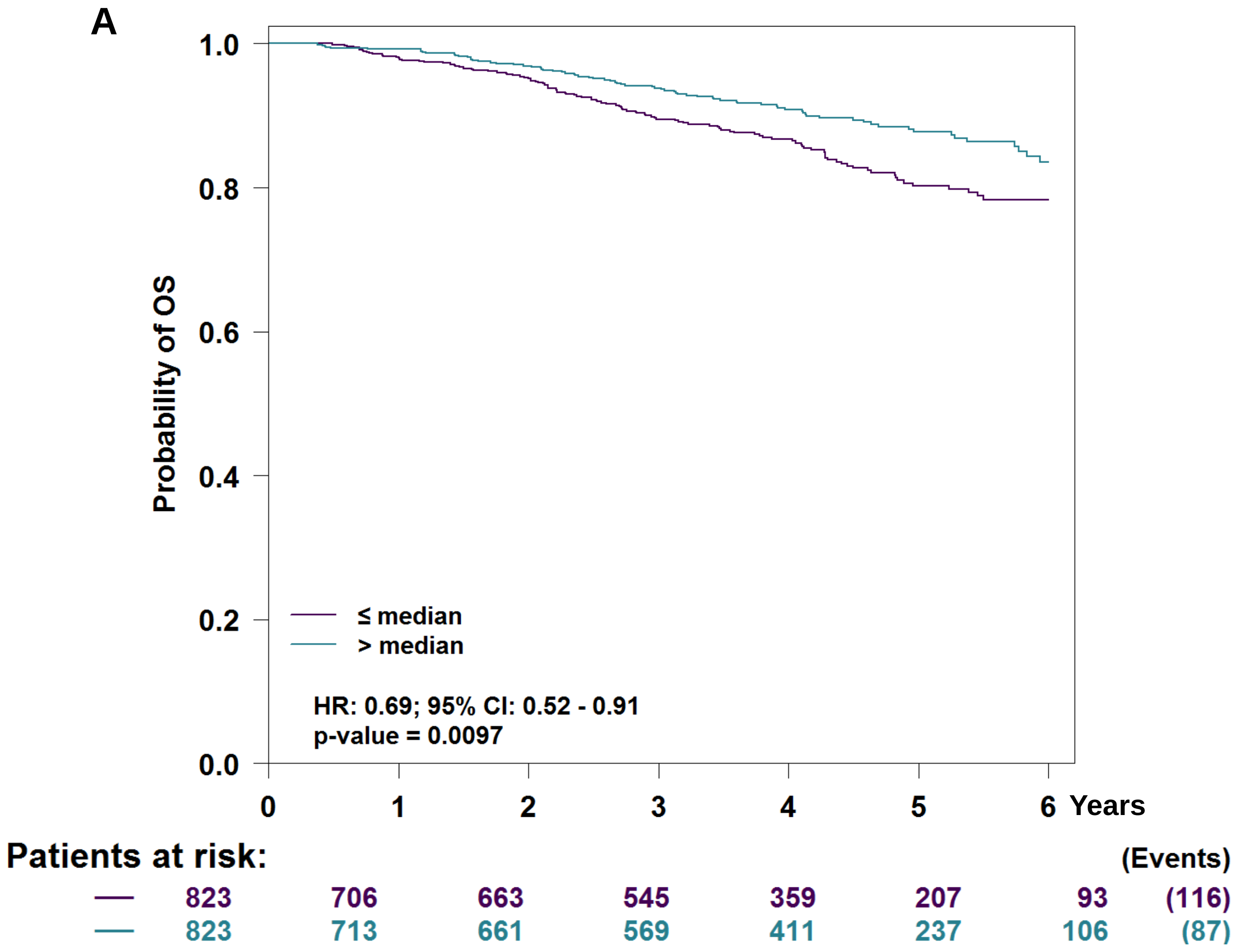

Figure S2. The decreasing of N6AMT1 mRNA level was significantly correlated with worse overall survival (OS) from the online BC (bc-GenExMiner v4.0).

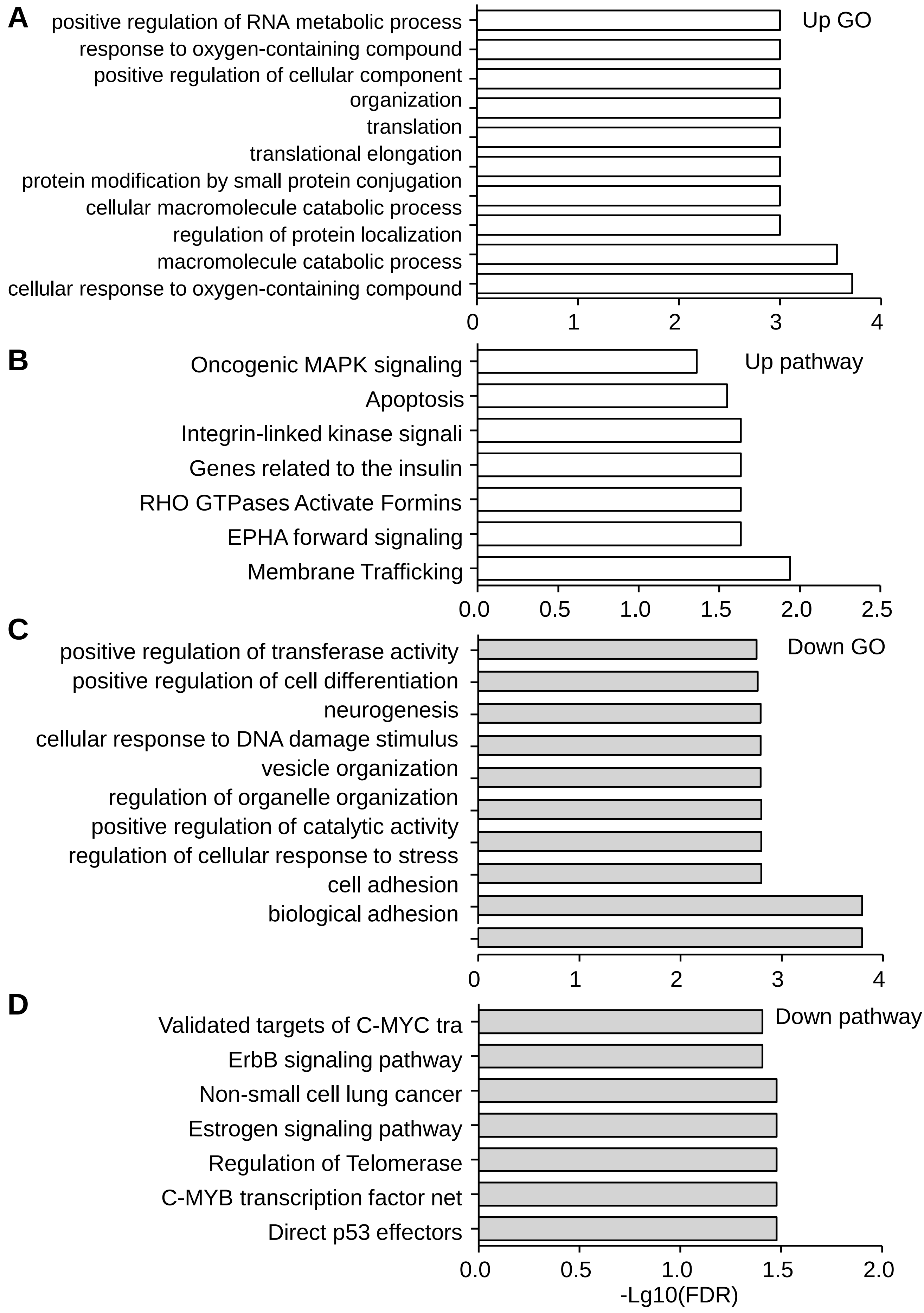
A
Up GO
positive regulation of RNA metabolic process
response to oxygen-containing compound
positive regulation of cellular component organization
translation
translational elongation
protein modification by small protein conjugation
cellular macromolecule catabolic process
regulation of protein localization
macromolecule catabolic process
cellular response to oxygen-containing compound
0
1
2
3
4
B
Up pathway
Oncogenic MAPK signaling
Apoptosis
Integrin-linked kinase signali
Genes related to the insulin
RHO GTPases Activate Formins
EPHA forward signaling
Membrane Trafficking
0.0
0.5
1.0
1.5
2.0
2.5
C
Down GO
positive regulation of transferase activity
positive regulation of cell differentiation
neurogenesis
cellular response to DNA damage stimulus
vesicle organization
regulation of organelle organization
positive regulation of catalytic activity
regulation of cellular response to stress
cell adhesion
biological adhesion
0
1
2
3
4
D
Down pathway
Validated targets of C-MYC tra
ErbB signaling pathway
Non-small cell lung cancer
Estrogen signaling pathway
Regulation of Telomerase
C-MYB transcription factor net
Direct p53 effectors
0.0
0.5
1.0
1.5
2.0
-Lg10(FDR)

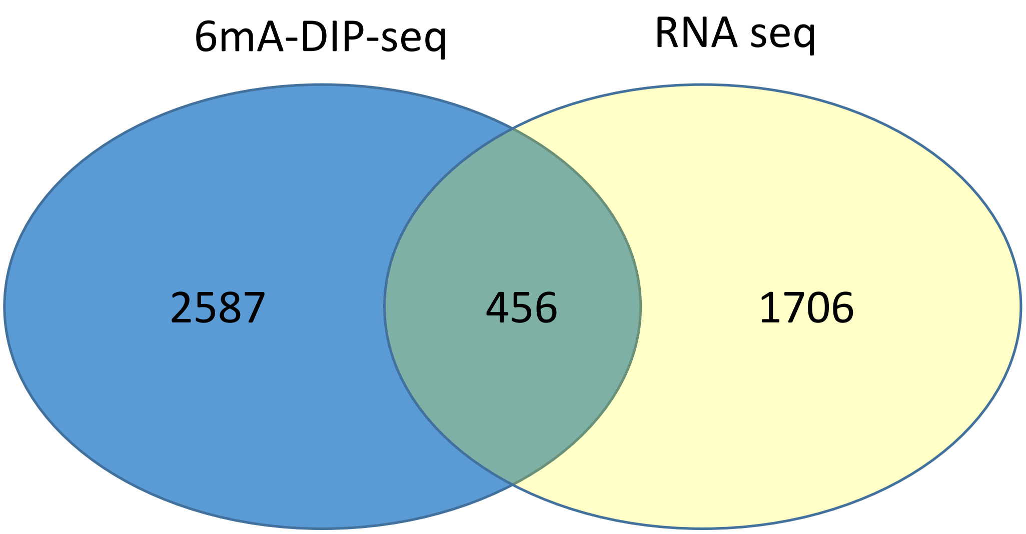
6mA-DIP-seq
RNA seq
2587
456
1706
