# Supplementary figures and images for: Reducing N6AMT1-mediated 6mA DNA modification promotes breast tumor progression via transcriptional repressing cell cycle inhibitors
Source: Cell Death Dis. 2022 Mar 7;13(3):216. doi: 10.1038/s41419-022-04661-8 (PMC8901905; doi:10.1038/s41419-022-04661-8)

# Uncropped figures for WB

**Figure 2A**

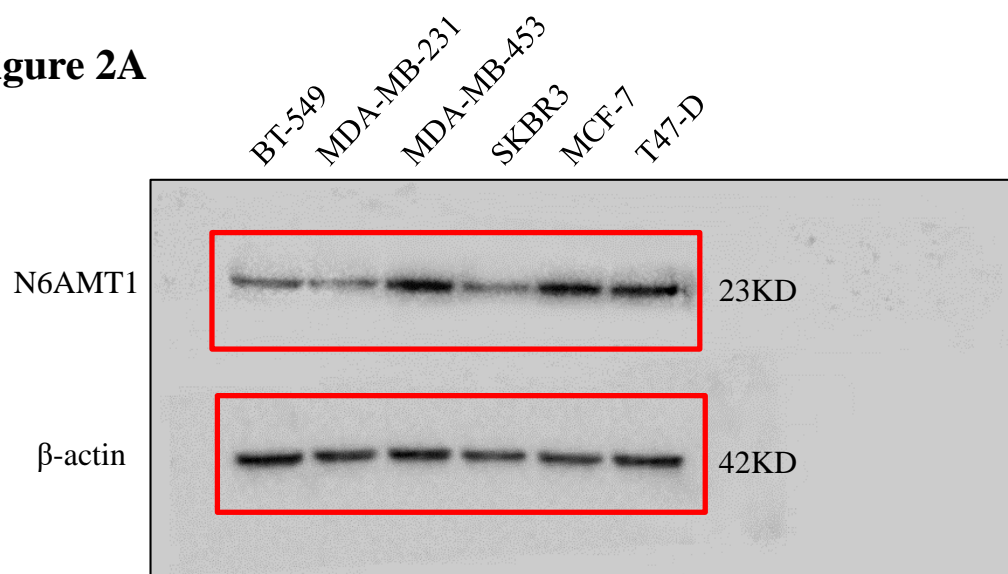

**Figure 2B**

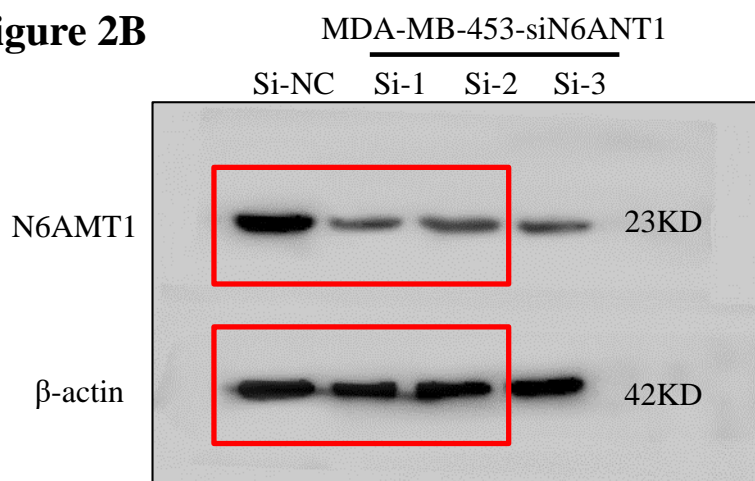

**Figure 2C**

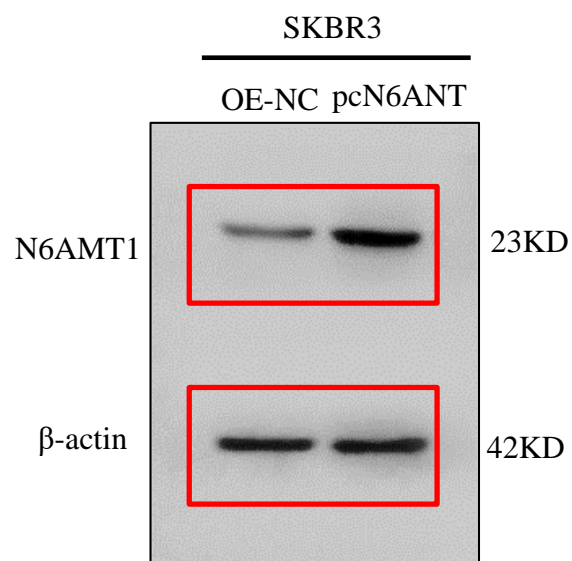

**Figure 3A**

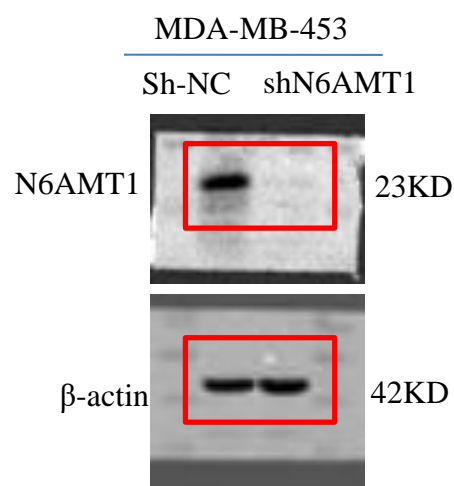

Supplement: Supplementary file 2 — Supplemental Material-Uncropped figures for WB [file 41419_2022_4661_MOESM2_ESM.pdf]
