# Supplementary material for: Reducing N6AMT1-mediated 6mA DNA modification promotes breast tumor progression via transcriptional repressing cell cycle inhibitors
Source: Cell Death Dis. 2022 Mar 7;13(3):216. doi: 10.1038/s41419-022-04661-8 (PMC8901905; doi:10.1038/s41419-022-04661-8)
Supplement: Supplementary file 3 — SUPPLEMENTAL Table S1 [file 41419_2022_4661_MOESM3_ESM.docx]

Table S1. Complete list of primers used in this work. Sequences are from the GenBank.

| Assay Name | Primer sequence (5’–3’) | | Products  (bp) |
| --- | --- | --- | --- |
|  | Forward: | Reverse: |  |
| **RT–qPCR** |  |  |  |
| N6AMT1 | GCAGGGGAGAACTTCGCTAC | CAGCGCGTTCAAAAGCAGAAA | 105 |
| RB1 | CCTCTCGTCAGGCTTGAGTT | ACAGATTCCCCACAGTTCCT | 180 |
| TP53 | TTCCTCCAACCAAGAACCAGA | GCTCAGTAGGTGACTCTTCACT | 140 |
| LATS2 | ACCCCAAAGTTCGGACCTTAT | CATTTGCCGGTTCACTTCTGC | 111 |
| TIPRL | GGCGTCCAAGACCCACATC | ACAGGCCACTTTAAGCATTCC | 199 |
| BTG1 | AGCGGATTGGACTGAGCAG | GGTGCTGTTTTGAGTGCTACC | 161 |
| REST | TCTCGGAGGTGGAGTACCTG | CCCAGTAAACGAGGTGACCAAA | 146 |
| MDM2 | TCGTCGGGTGAGGGTACTG | AACCACTTCTTGGAACCAGGT | 152 |
| P21 | AGTCAGTTCCTTGTGGAGCC | CATTAGCGCATCACAGTCGC | 184 |
| β-ACTIN | CACCAACTGGGACGACAT | ACAGCCTGGATAGCAACG | 188 |
| **6mA-IP-qPCR** | |  |  |
| RB1 | GCTAGCAAGGCTTCTGTAGGT | TCAACTTTCTAGAAGTGTGGGCA | 298 |
| TP53 | ATGGACAGACACTGCCTTCTG | GCAGGGTGCTAAACACGTAGA | 125 |
| LATS2 | TGCTCCTGCAAACTAGACCTG | GACTCATTTGCTTCCAGTGCC | 142 |
| TIPRL | GGTGCAGTATTTCTGGGGCT | CACGACCTTGACTCTGACCC | 355 |
| BTG1 | ACACACTGCCAGCCTACTTC | GGCTTAAGAGCTGCACGAGA | 206 |
| REST | GAGAGGCTGACTTGCCTGAT | TGGCTGTCAACTTCCAGCTT | 286 |
| MDM2 | AAGTGCTGGCAAACAATGGC | AGCAACACTCTTGGTTGATGT | 374 |
| P21 | TTCACTGGGCCAACCACATC | CTCACACTTGGGTCTCCTCT | 101 |
